# An inverted U-shaped association between high-sensitivity C-reactive protein and the albumin ratio and hepatic steatosis and liver fibrosis: a population-based study

**DOI:** 10.3389/fnut.2025.1534200

**Published:** 2025-04-15

**Authors:** Xiaorong Ma, Baoyu Li, Yuwei Liu, Xiaoyan Guo

**Affiliations:** Department of Gastroenterology, The Second Affiliated Hospital of Xi'an Jiaotong University, Xi'an, Shaanxi, China

**Keywords:** the ratio of high-sensitivity C-reactive protein to albumin, NAFLD, NHANES, hepatic steatosis, liver fibrosis

## Abstract

**Background:**

The high-sensitivity C-reactive protein to albumin (CAR) ratio is a comprehensive measure of inflammation *in vivo*. Hepatic steatosis and fibrosis are significantly correlated with inflammation. The present study aimed to explore the possible associations between CAR and hepatic steatosis and fibrosis in the American population.

**Methods:**

The study population involved the National Health and Nutrition Examination Survey (NHANES) participants from 2017 to 2020. The natural logarithm of CAR, calculated as Ln(CAR) with base “e,” was used for further analyses. The relationships between Ln(CAR) and the controlled attenuation parameter (CAP) and between Ln(CAR) and liver stiffness measurement (LSM) were investigated through multivariate linear regression analysis. Interaction and subgroup analysis identified factors affecting these variables. Nonlinear relationships were elucidated by smoothing curves and threshold effect analysis. Receiver operating characteristic (ROC) curve analysis was performed to evaluate the predictive performance of the CAR for non-alcoholic fatty liver disease (NAFLD). The results were adjusted for U.S. population estimates.

**Results:**

The study included a total of 7,404 individuals. Ln(CAR) was positively correlated with CAP in the fully adjusted model, with an effect value of *β* = 1.827 (95% CI, 0.611, 3.042). A more pronounced positive association was observed among participants with a BMI ≥ 25 kg/m^2^ in the subgroup analysis. An inverted U-shaped association was shown between Ln(CAR) and CAP through smooth curve fitting and a two-segment linear regression model, with an inflection point of (−9.594). ROC curve analysis showed that CAR had a moderate predictive value for NAFLD (AUC = 0.6895), with a sensitivity of 0.7276 and a specificity of 0.6092. No significant association was detected between Ln(CAR) and the LSM.

**Conclusion:**

We demonstrate an inverted U-shaped relationship between Ln(CAR) and CAP risk within the U.S. demographic. Our results suggest that CAR may serve as a valuable diagnostic tool for NAFLD. Further prospective research is necessary to validate this conclusion.

## 1 Introduction

Nonalcoholic fatty liver disease (NAFLD) is an escalating public health concern (1) and is anticipated to emerge as the predominant driver contributing to cirrhosis and primary liver cancer worldwide (2, 3). The primary characteristic of NAFLD is extensive hepatic fat deposition, with insulin resistance generally observed as a concurrent feature (4). It is defined as fat accumulation in more than 5% of liver parenchymal cells histologically, following the exclusion of other well-established etiologies of liver fat infiltration (4). The pathological spectrum of NAFLD spans a continuum of conditions, including simple steatosis, nonalcoholic steatohepatitis (NASH), and advanced stages such as fibrosis and cirrhosis (5). Vibration-controlled transient elastography (VCTE) is an effective diagnostic technique, that has been proven to measure the controlled attenuation parameter (CAP) and liver stiffness measurement (LSM) noninvasively, thus accurately evaluating the degree of liver steatosis and fibrosis (6). To date, transient elastography has been widely used for patients with NAFLD because of its irreplaceable advantages (7–9).

C-reactive protein and albumin are both acute-phase reactive proteins synthesized by the liver, with C-reactive protein levels increasing and albumin levels typically decreasing during the course of infection or inflammation (10). Nevertheless, high-sensitivity C-reactive protein (hs-CRP) serves as an indicator of low-grade systemic inflammation that can be consistently evaluated (11). The ratio of hs-CRP-to-albumin (CAR) integrates two commonly measured biomarkers, providing a comprehensive picture of inflammation and nutritional status. Evidence has identified it as a valuable quantitative marker for assessing the inflammatory load (12). Currently, CAR is an established prognostic biomarker in solid tumors, with particular relevance to gastrointestinal cancers (13–19) and respiratory cancers (20–22). Additionally, its prognostic value has been investigated in gynecological malignancies (23, 24). Furthermore, high CAR values also predict a poor prognosis for cardiovascular disease (25) and critical coronavirus disease 2019 (COVID-19) (26, 27). Existing evidence supports the close relationship between inflammation and the development and progression of NAFLD (28–30). The dysregulated recruitment of immune cells and the extensive release of inflammatory mediators are essential links leading to liver damage, driving the development of hepatic steatosis and fibrosis (31–34). A recent study (35) investigated the relationship between the systemic inflammation index (SII) and the severity of hepatic steatosis and liver cirrhosis, revealing a significant statistical correlation. However, there has been no study on the relationship between the CAR and NAFLD yet.

Accordingly, for the purpose of clarifying the prospective associations between CAR and CAP and LSM, we downloaded the National Health and Nutrition Examination Survey (NHANES) metadata from 2017 to 2020. The subjects included individuals ranging from 12 to 80 years of age.

## 2 Materials and methods

### 2.1 Data source and study population

**Figure 1:**
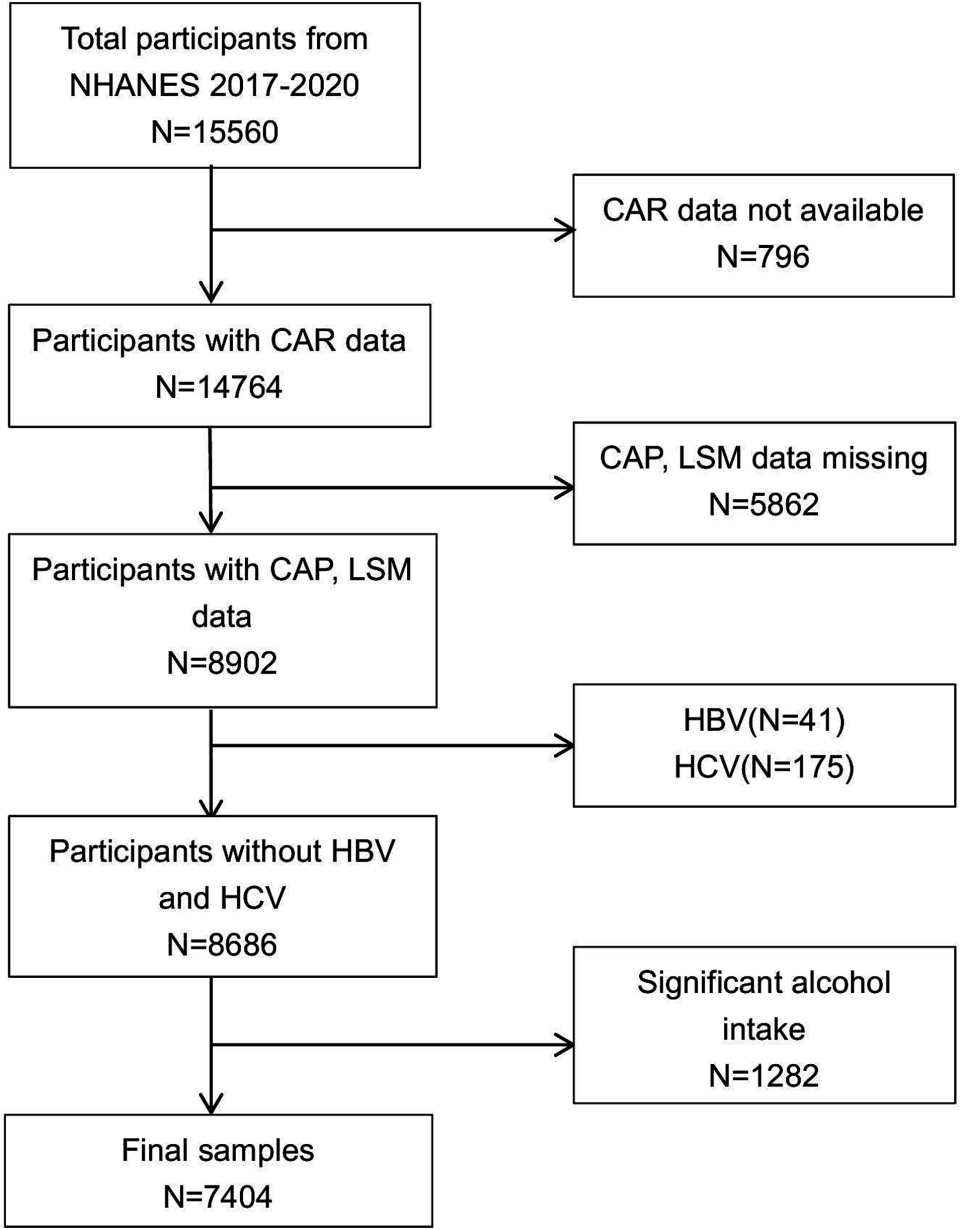
Flowchart of participant selection. NHANES, National Health and Nutrition Examination Survey.

Administered by the National Center for Health Statistics (NCHS), the National Health and Nutrition Examination Survey (NHANES) gathers comprehensive data on the health and nutritional profiles of a representative sample of the U.S. non-institutionalized population through structured interviews, clinical assessments, and laboratory analyses. All individuals acknowledged their understanding and provided written consent. We included an initial cohort of 15,560 participants from the NHANES 2017–2020 dataset. Then, participants missing CAR (*n* = 796), CAP and LSM data (*n* = 5,862), positive serological markers for HBV (*n* = 41) or HCV (*n* = 175), with a history of excessive alcohol consumption (*n* = 1,282) were excluded. After exclusion, the final study included 7,404 participants (Figure 1).

### 2.2 Dependent and independent variables

We set CAR as an independent variable, with CAP and LSM as dependent variables. High-sensitivity C-reactive protein (hs-CRP) (mg/L) and albumin (g/L), which are components of the independent variables, were determined via a latex particle-enhanced immunoturbidimetric assay kit from Roche Diagnostics (Indianapolis), and data were finally read using a Roche Modular c501 Chemistry analyzer (Roche Diagnostics). Furthermore, the median values of the CAP and LSM served as dependent variables and were used as objective measures to stage hepatic steatosis and fibrosis. The Fibroscan^®^ 502 Touch was operated by trained technical staff for the measurement of the LSM and CAP. A recent study published in the *Journal of Hepatology* indicated that the best cutoff point and 95% CI of 248 dB/m (237–261), 268 dB/m (257–284), and 280 dB/m (268–294) are reliable for determining steatosis grades exceeding S0, S1, and S2, respectively (36). Additionally, data derived from a meta-analysis comprising 37 primary studies supported an LSM threshold of <10.0 kPa to exclude advanced fibrosis and ≥20.0 kPa for the preliminary confirmation of cirrhosis (37).

### 2.3 Covariates

Theoretically, any variable potentially influencing CAR, CAP, and LSM levels in a multiple linear regression model should be included as a covariate for adjustment. However, it is nearly impossible for us to fulfill such study conditions completely. Consequently, referring to established authoritative research, we adopted widely recognized variables as covariates throughout our analysis (38–40).

In this study, variables were initially categorized into demographic characteristics, including age, sex, ethnicity, poverty income ratio, and educational level. Subsequently, behavioral factors, such as levels of moderate physical activity, smoking behavior, and alcohol consumption, were assessed in detail. Finally, data on clinical factors—including body mass index (BMI), liver enzyme levels, blood lipid levels, uric acid, total calcium, serum phosphorus, and notable comorbidities such as hypertension, diabetes, and a confirmed record of cardiovascular disease—were collected to account for their potential confounding effects.

Hypertension was defined according to the following criteria: (1) self-reported history of a physician-diagnosed hypertension; (2) an average systolic blood pressure (SBP) of ≥140 mmHg and/or diastolic blood pressure (DBP) of ≥90 mmHg, based on three consecutive measurements; (3) affirmative responses to any of the following survey questions: “Ever told had high blood pressure—2 or more times, “Currently taking prescribed medication for hypertension,” or “Now taking prescribed medicine for high blood pressure (HBP).”

Diabetes mellitus (DM) was defined according to the following criteria: (1) self-reported physician diagnosis of diabetes; (2) glycated hemoglobin (HbA1c) level of ≥6.5%; (3) fasting plasma glucose (FPG) concentration of ≥126 mg/dL (7.0 mmol/L); (4) random blood glucose level of ≥200 mg/dL (11.1 mmol/L); (5) affirmative responses to any of the following questionnaire items: “Ever told by a doctor that you have diabetes,” “Currently taking insulin,” or “Taking oral medication to lower blood sugar.”

History of cardiovascular disease (CVD) was defined based on self-reported physician diagnoses from the Medical Condition questionnaire. Participants were classified as having a history of CVD if they provided affirmative responses to any of the following items: “Ever been told by a doctor that you had congestive heart failure,” “Ever been diagnosed with coronary heart disease,” “Ever been told you had angina or angina pectoris,” “Ever been diagnosed with a heart attack,” or “Ever been told you had a stroke.”

### 2.4 Statistical analysis

The data were analyzed and visualized using R statistical software (version 4.2.0) and EmpowerStats (version 6.0). Given the nonnormal distribution of the CAR data, a logarithmic transformation with the base “e” was applied, and the transformed values were subsequently used in all analyses conducted in this study. Categorical variables are expressed as percentages (%), while continuous variables are presented as means ± standard errors (SE) for normally distributed or approximately normally distributed data, and as medians with interquartile ranges (IQRs) for non-normally distributed data. Weighted multiple linear regression analysis was employed to examine the relationships between Ln(CAR) and CAP and LSM, estimating beta coefficients (*β*) and 95% confidence intervals (CIs). Three distinct models were constructed according to the covariates: Model 1, which was non-adjusted; Model 2, which was adjusted for age, sex, and race; and Model 3, which was adjusted for all the covariates outlined in Table 1. Additionally, subgroup analysis was performed on the basis of the BMI (kg/m^2^) categories (<25, 25–30, ≥30) of the participants to identify specific cohort characteristics requiring further attention. Smoothing curves were used to illustrate the shape of the relationship between the Ln(CAR) and CAP and between the Ln(CAR) and the LSM. Using a threshold regression model, the inflection point for Ln(CAR) on the smoothing curve was estimated. The ROC curve was employed to compare the diagnostic accuracy of CAR and SII in detecting NAFLD. The diagnostic performance of NAFLD was evaluated by calculating the area under the curve (AUC). *p* < 0.05 was considered to indicate statistical significance.

**Table 1:** Weighted characteristics of the study population on the basis of the controlled attenuation parameter (CAP).

Study variables	Non-NAFLD (CAP < 248)	Mildly NAFLD (248 ≤ CAP < 268)	Moderate NAFLD (268 ≤ CAP < 280)	Severe NAFLD (CAP ≥ 280)	*p* value
	*n* = 3,469	*n* = 781	*n* = 531	*n* = 2,623	
Age (years)	39.603 ± 20.490	47.762 ± 19.736	49.911 ± 19.109	50.809 ± 17.561	<0.001
Gender (%)					<0.001
Men	43.726	46.405	50.427	54.913	
Women	56.274	53.595	49.573	45.087	
Race(%)					<0.001
Non-Hispanic White	61.096	61.627	62.593	62.685	
Non-Hispanic Black	13.528	9.900	10.640	9.419	
Mexican American	7.072	7.769	9.062	10.797	
Other Race	18.304	20.704	17.705	17.099	
Education level (%)					<0.001
Never attended high school	8.971	16.386	12.884	10.927	
High school and above	91.029	83.614	87.116	89.073	
Income to poverty ratio	3.086 ± 1.603	3.029 ± 1.526	3.088 ± 1.606	3.028 ± 1.557	0.477
BMI (kg/m^2^)	25.058 ± 5.081	28.625 ± 5.974	29.999 ± 5.405	33.869 ± 7.310	<0.001
Moderate activities (%)					0.017
Yes	45.904	47.931	54.110	46.408	
No	54.096	52.069	45.890	53.592	
Smoked at least 100 cigarettes					<0.001
Yes	34.628	44.523	37.100	38.777	
No	65.372	55.477	62.900	62.223	
DM	4.285	10.667	12.535	26.206	<0.001
Hypertension	18.342	32.282	34.966	48.048	<0.001
History of CVD	7.805	9.312	5.708	8.116	0.124
Laboratory features					
ALT (IU/L)	14.000 (8.000)	16.000 (10.000)	17.000 (10.000)	21.000 (15.000)	<0.001
AST (IU/L)	18.000 (6.000)	18.000 (7.000)	18.000 (10.000)	20.000 (9.000)	<0.001
ALP (IU/L)	77.000 (40.000)	78.000 (36.000)	78.000 (32.000)	79.000 (31.000)	<0.001
LDL-C (mmol/L)	2.642 ± 0.601	2.731 ± 0.679	2.752 ± 0.661	2.744 ± 0.680	<0.001
HDL-C (mmol/L)	1.488 ± 0.381	1.038 ± 0.602	1.194 ± 1.438	1.248 ± 0.875	<0.001
TG (mmol/L)	0.898 ± 0.331	1.157 ± 0.651	1.384 ± 1.455	1.561 ± 0.966	<0.001
TC (mmol/L)	4.607 ± 1.017	4.763 ± 1.025	4.962 ± 1.110	4.856 ± 1.068	<0.001
Total calcium (mmol/L)	2.334 ± 0.089	2.327 ± 0.095	2.335 ± 0.092	2.328 ± 0.091	0.018
Serum phosphorus (mmol/L)	1.206 ± 0.193	1.183 ± 0.193	1.183 ± 0.181	1.152 ± 0.181	<0.001
UC (mmol/L)	290.903 ± 76.379	314.522 ± 75.116	317.176 ± 77.478	343.496 ± 86.398	<0.001
CAR (10^-5^)	2.300 (4.900)	3.900 (8.100)	4.4.00 (7.700)	6.900 (11.800)	<0.001
Ln(CAR)	−10.529 ± 1.186	−10.164 ± 1.241	−10.053 ± 1.047	−9.605 ± 1.104	<0.001
LSM (kPa)	4.600 (1.700)	4.800 (1.700)	5.000 (1.800)	5.600 (2.600)	<0.001

Mean ± SD for continuous variables: *p* values were calculated via a weighted linear regression model. % for categorical variables: *p* values were calculated via the weighted chi-square test. BMI, body mass index; DM, diabetes mellitus; LDL-C, low-density lipoprotein cholesterol; HDL-C, high-density lipoprotein cholesterol; ALT, alanine transaminase; ALP, alkaline phosphatase; AST, aspartate aminotransferase; TG, triglyceride; TC, total cholesterol; UC, uric acid; LSM, liver stiffness measure; CAP, controlled attenuation parameter; CAR, ratio of high-sensitivity C-reactive protein to albumin; Ln(CAR), with “e” as the base, the logarithm of CAR.

## 3 Results

### 3.1 Baseline demographic and clinical features

In accordance with stringent inclusion and exclusion criteria, 3,597 men (48.58%) and 3,807 women (51.42%) were enrolled in this study. Among these participants, the average age was 45.10 ± 21.39 years. The racial distribution consisted of 34.16% non-Hispanic White, 25.63% non-Hispanic Black, 11.90% Mexican American, and 28.31% from other racial backgrounds. For Ln(CAR), CAP, and LSM, the average (SD) values were (−10.12) (1.27), 257.25 (63.17) dB/m, and 5.84 (4.92) kPa, respectively.

Table 1 shows the baseline demographic and clinical characteristics of all participants stratified by CAP. Variables such as age, sex, racial background, education level, moderate activities, smoking status, diabetes, and hypertension status were significantly different between the severe NAFLD and the non-NAFLD cohorts. In the severe NAFLD group, elevated levels of age, BMI, alanine transaminase (ALT), aspartate aminotransferase (AST), uric acid, low-density lipoprotein cholesterol (LDL-C), triglyceride (TG), total cholesterol (TC), Ln(CAR), and LSM were observed, while serum phosphorus, high-density lipoprotein cholesterol (HDL-C), alkaline phosphatase (ALP), and total calcium were notably lower.

Table 2 shows the baseline demographic and clinical characteristics of all participants stratified by LSM. In patients with cirrhosis, analogous patterns were observed when contrasted with those lacking evidence of advanced liver fibrosis, with significant differences noted in age, sex, educational level, BMI, diabetes, hypertension, ALT, AST, HDL-C, uric acid, total calcium, serum phosphorus, Ln(CAR), and LSM. However, the comparison between these groups revealed no substantial variations in TG, LDL-C, and ALP levels (*p* > 0.05).

**Table 2:** Weighted characteristics of the study population on the basis of the median liver stiffness measurement (LSM).

Study variables	Advanced liver fibrosis		Cirrhosis	*p* value
	No (LSM < 10)	Yes(10 ≤ LSM < 20)	(LSM ≥ 20)	
	*n* = 7,141	*n* = 158	*n* = 105	
Age(years)	44.986 ± 20.056	52.683 ± 17.521	50.769 ± 17.850	<0.001
Gender(%)				0.004
Men	48.225	51.560	64.664	
Women	51.775	48.44	35.336	
Race(%)				0.486
Non-Hispanic White	61.666	69.356	61.903	
Non-Hispanic Black	11.527	8.377	11.676	
Mexican American	8.649	9.350	6.012	
Other Race	18.158	12.917	20.409	
Education level (%)				<0.001
Never attended high school	10.254	13.14	8.973	
High school and above	89.746	86.86	91.027	
Income to poverty ratio	3.067 ± 1.580	2.976 ± 1.568	2.600 ± 1.409	0.011
BMI (kg/m^2^)	28.588 ± 6.860	38.088 ± 8.230	41.376 ± 12.555	<0.001
Moderate activities (%)				0.091
Yes	46.905	40.281	58.501	
No	53.095	59.719	41.499	
Smoked at least 100 cigarettes				0.366
Yes	37.250	39.218	49.823	
No	62.750	60.782	50.177	
DM	12.465	42.343	38.689	<0.001
Hypertension	30.729	63.395	51.116	<0.001
History of CVD	7.886	9.122	8.984	0.790
Laboratory features				
ALT (IU/L)	16.000 (11.000)	21.000 (18.000)	19.000 (88.000)	<0.001
AST (IU/L)	19.000 (7.000)	21.000 (12.000)	21.000 (14.000)	<0.001
ALP (IU/L)	78.000 (35.000)	83.000 (38.000)	87.000 (38.000)	0.526
LDL-C (mmol/L)	2.697 ± 0.645	2.722 ± 0.669	2.595 ± 0.623	0.260
HDL-C (mmol/L)	1.387 ± 0.383	1.238 ± 0.387	1.202 ± 0.344	<0.001
TG (mmol/L)	1.056 ± 0.735	1.171 ± 0.506	1.162 ± 0.801	0.058
TC (mmol/L)	4.742 ± 1.051	4.706 ± 1.073	4.476 ± 1.012	0.041
Total calcium (mmol/L)	2.331 ± 0.090	2.334 ± 0.105	2.307 ± 0.083	0.029
Serum phosphorus (mmol/L)	1.184 ± 0.190	1.148 ± 0.179	1.147 ± 0.157	0.010
UC (mmol/L)	312.529 ± 82.782	356.213 ± 94.590	368.665 ± 82.942	<0.001
CAR (10^-5^)	3.600 (7.800)	8.400 (13.400)	13.100 (18.000)	<0.001
Ln(CAR)	−10.158 ± 1.222	−9.362 ± 0.912	−8.864 ± 0.879	<0.001
CAP (dB/m)	256.093 ± 61.259	329.186 ± 61.977	328.418 ± 62.723	<0.001

Mean ± SD for continuous variables: *p* values were calculated via a weighted linear regression model. % for categorical variables: *p* values were calculated via the weighted chi-square test. BMI, body mass index; DM, diabetes mellitus; LDL-C, low-density lipoprotein cholesterol; HDL-C, high-density lipoprotein cholesterol; ALT, alanine transaminase; ALP, alkaline phosphatase; AST, aspartate aminotransferase; TG, triglyceride; TC, total cholesterol; UC, uric acid; LSM, liver stiffness measure; CAP, controlled attenuation parameter; CAR, ratio of high-sensitivity C-reactive protein to albumin; Ln(CAR), with e as the base, the logarithm of CAR.

### 3.2 Associations between Ln(CAR) and the controlled attenuation parameter

Table 3 provides a summary of the outcomes from the multiple linear regression analysis. The unadjusted model indicated a positive correlation between Ln(CAR) and CAP, and this association was still robust after adjusting for all potential confounding variables. Additionally, for each unit of Ln(CAR) increase, CAP increased by 1.83 (*β* = 1.83, 95% CI 0.611, 3.042) units in the fully adjusted model. The Ln(CAR) was subsequently divided into tertiles for analysis. An increasing trend was statistically significant (*p* for trend < 0.001) concerning the relationship between Ln(CAR) and CAP, as Ln(CAR) levels rose. Compared with Tertile 1, Tertile 3 exhibited the greatest effect size, with a statistically significant difference (*β* = 6.68, 95% CI: 3.191, 10.171).

**Table 3:** Association between Ln(CAR) and CAP.

Ln(CAR)	Model 1 β (95% CI) *p* value	Model 2 β (95% CI) *p* value	Model 3 β (95% CI) *p* value
CAP(dB/m)			
Continuous	18.484 (17.999,18.970) <0.001	17.423 (16.944, 17.903) <0.001	1.827 (0.611, 3.042) 0.003
Categories			
Tertile 1	Reference	Reference	Reference
Tertile 2	32.426 (29.169, 35.683) <0.001	26.724 (23.564, 29.884) <0.001	6.947 (3.902, 9.992) <0.001
Tertile 3	54.862 (51.569, 58.154) <0.001	51.598 (48.375, 54.822) <0.001	6.681 (3.191, 10.171) <0.001
*p* for trend	<0.001	<0.001	<0.001

Model 1: no covariates were adjusted. Model 2: Age, gender, and race were adjusted. Model 3: Age, gender, race, educational level, BMI, income-to-poverty ratio, moderate activities, smoking status, DM, hypertension, history of CVD, ALP, ALT, AST, total calcium, TC, TG, LDL-C, HDL-C, UC, and serum phosphorus were adjusted. NAFLD, nonalcoholic fatty liver disease; CAP, controlled attenuation parameter; LSM, liver stiffness measure; CAR, high-sensitivity C-reactive protein to albumin ratio.

Table 4 shows the subgroup analysis and interaction results stratified according to BMI. Analysis revealed a notable interaction effect of BMI and Ln(CAR) on CAP (*p* for interaction = 0.011), indicating that the effect of Ln(CAR) on CAP differed according to BMI level. Specifically, among individuals categorized as overweight [3.094, (1.080, 5.108)] and obese [2.497, (0.570, 4.425)], a stronger positive association was observed between Ln(CAR) and CAP than in subjects with a normal weight, where the association was weaker and nonsignificant [−0.093, (−2.955, 1.150)]. The interaction suggested that the effect of Ln(CAR) on CAP was notably more pronounced among individuals with higher BMIs.

**Table 4:** Subgroup analysis of the associations between Ln(CAR) and CAP.

CAP(dB/m)		
Subgroups	β (95% CI) *p* value	*p* for interaction
BMI (kg/m^2^)		0.011
<25	−0.903 (−2.955, 1.150) 0.389	
≥25, <30	3.094 (1.080, 5.108) 0.003	
≥30	2.497 (0.570, 4.425) 0.011	

Age, gender, race, educational level, BMI, income-to-poverty ratio, moderate activities, smoking status, DM, hypertension, history of CVD, ALP, ALT, AST, total calcium, TC, TG, LDL-C, HDL-C, UC, and serum phosphorus were adjusted. NAFLD, nonalcoholic fatty liver disease; CAP, controlled attenuation parameter; LSM, liver stiffness measure; CAR, high-sensitivity C-reactive protein to albumin ratio.

**Figure 2:**
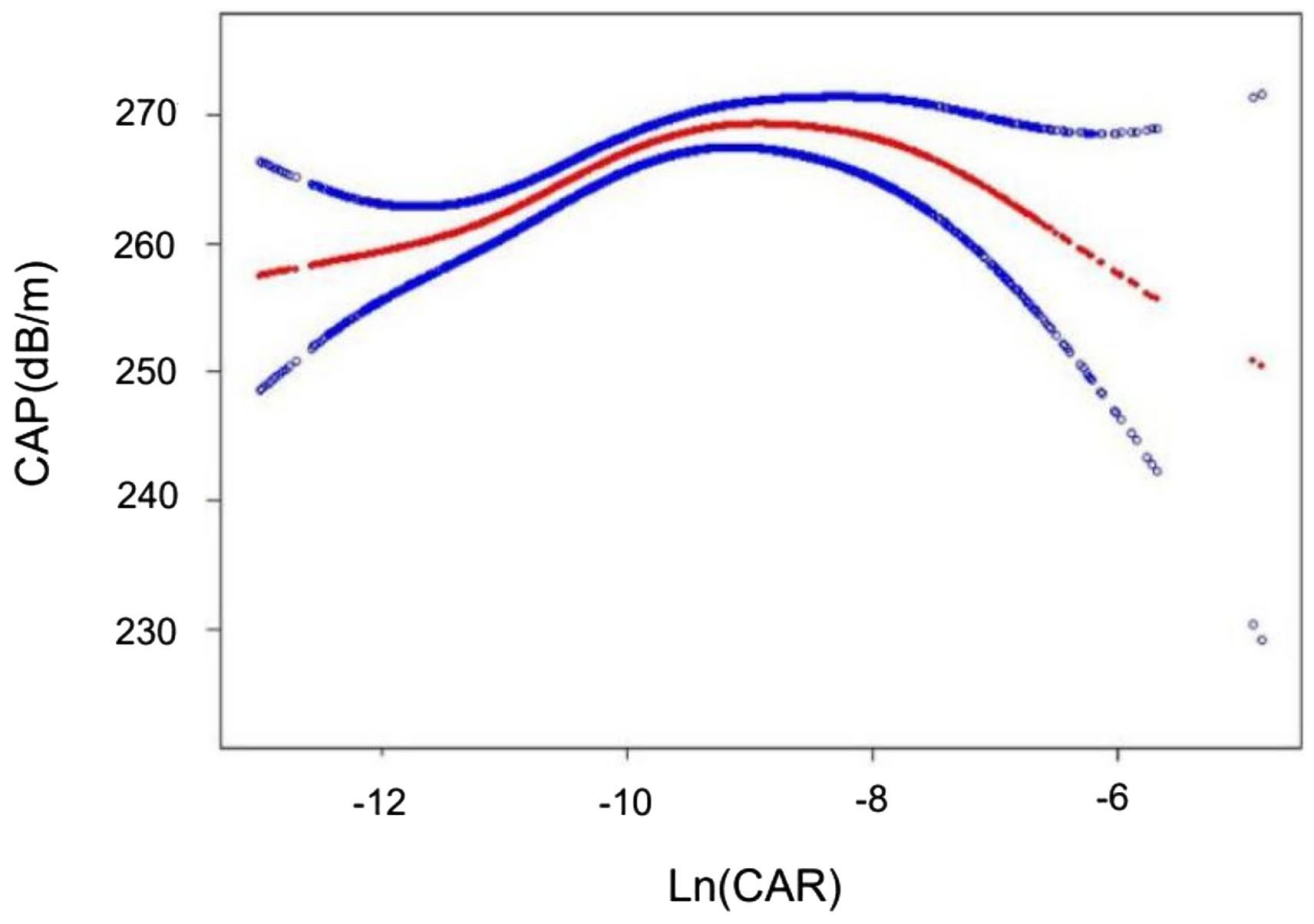
The inverted U-shaped curve associations between Ln(CAR) and CAP. The solid red line represents the smooth curve fit between variables. The blue bands represent the 95% confidence interval from the fit.

The association of Ln(CAR) with CAP was identified as a nonlinear, inverted U-shaped pattern through smooth curve fitting (Figure 2). A two-segment linear regression model was subsequently employed to estimate the breakpoint (K), which was determined to be (−9.594). An effect size of 3.657 was detected to the left of K, with a 95% CI ranging from 1.804–5.510. In contrast, to the right of the K, the effect size was −0.957, with the 95% CI ranging from −3.406 to 1.493, suggesting a reversal of the trend observed on the left side (Table 5). That said, we found no correlation between Ln(CAR) and CAP when (K) > (−9.594), while a positive relationship was detected when K < (−9.594).

**Table 5:** Threshold effect analysis of Ln(CAR) on CAP using a two-piecewise linear regression model.

CAP (dB/m)	Adjusted β (95% CI) *p* value
Ln(CAR)	
Linear effect	1.827 (0.611, 3.042) 0.003
Inflection point	−9.594
Ln(CAR) < (−9.594)	3.657 (1.804, 5.510) <0.001
Ln(CAR) > (−9.594)	−0.957 (−3.406, 1.493) 0.443
Log likelihood ratio	0.010

Age, gender, race, educational level, BMI, income-to-poverty ratio, moderate activities, smoking status, DM, hypertension, history of CVD, ALP, ALT, AST, total calcium, TC, TG, LDL-C, HDL-C, UC, and serum phosphorus were adjusted. CAP, controlled attenuation parameter; CAR, high-sensitivity C-reactive protein to albumin ratio.

### 3.3 CAR as a predictor of NAFLD: ROC analysis

**Figure 3:**
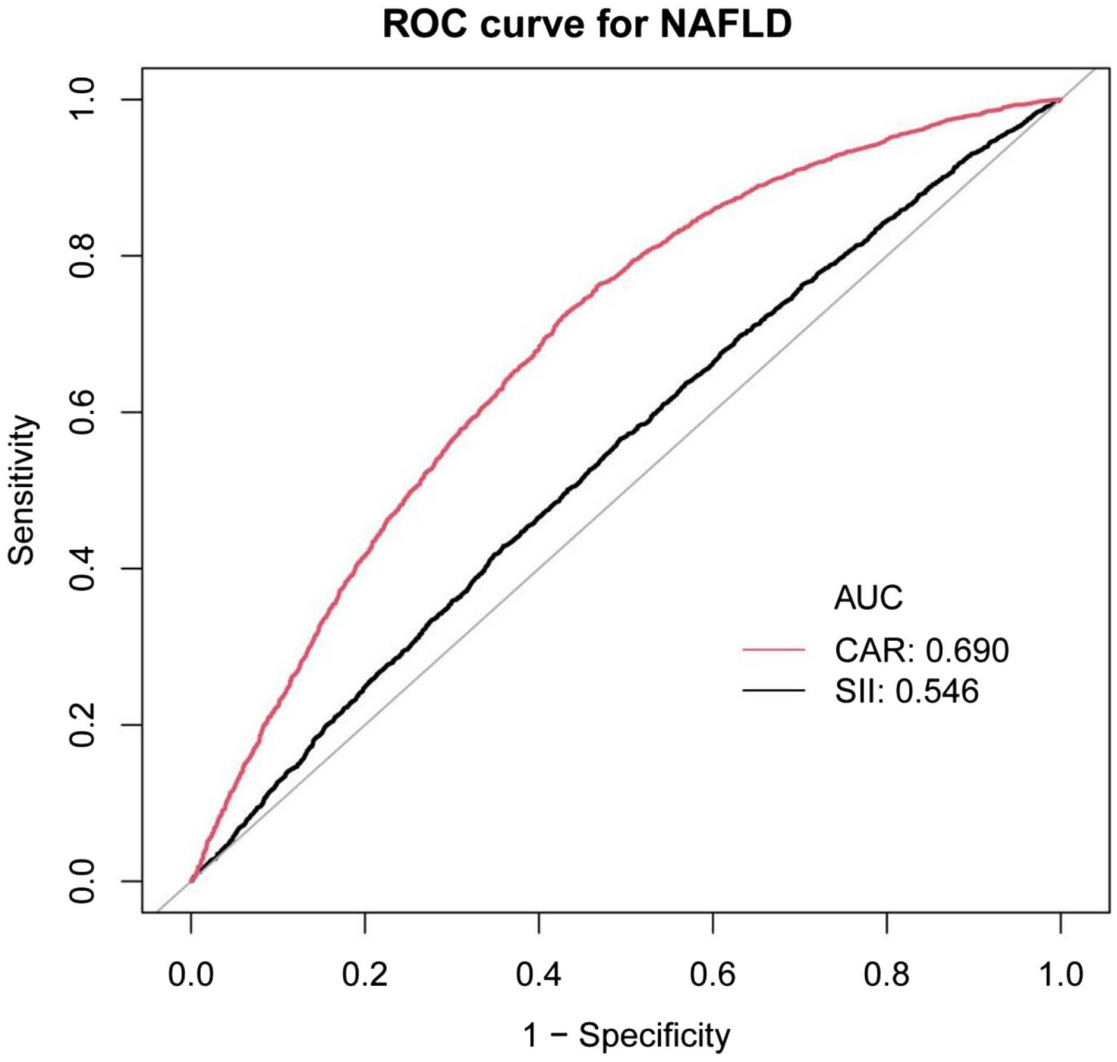
ROC curves for CAR, compared to SII for NAFLD onset. As determined by AUC, the predictive value for CAR is more significant than SII.

Previous studies have demonstrated a significant positive correlation between systemic immune-inflammation index (SII), a valuable systemic inflammation index, and CAP. This finding has been insightful for our research. Therefore, in this study, we compared CAR with SII using ROC curve analysis. The ROC curves, presented in Figure 3, reveal that the area under the curve (AUC) for CAR is significantly superior to that of SII. As shown in Supplementary Table 1, the AUC for CAR was 0.6895, with a sensitivity of 0.7276 and a specificity of 0.6092 in predicting NAFLD. Compared to SII, CAR exhibited notably higher sensitivity and specificity.

### 3.4 Associations between Ln(CAR) and the LSM

**Figure 4:**
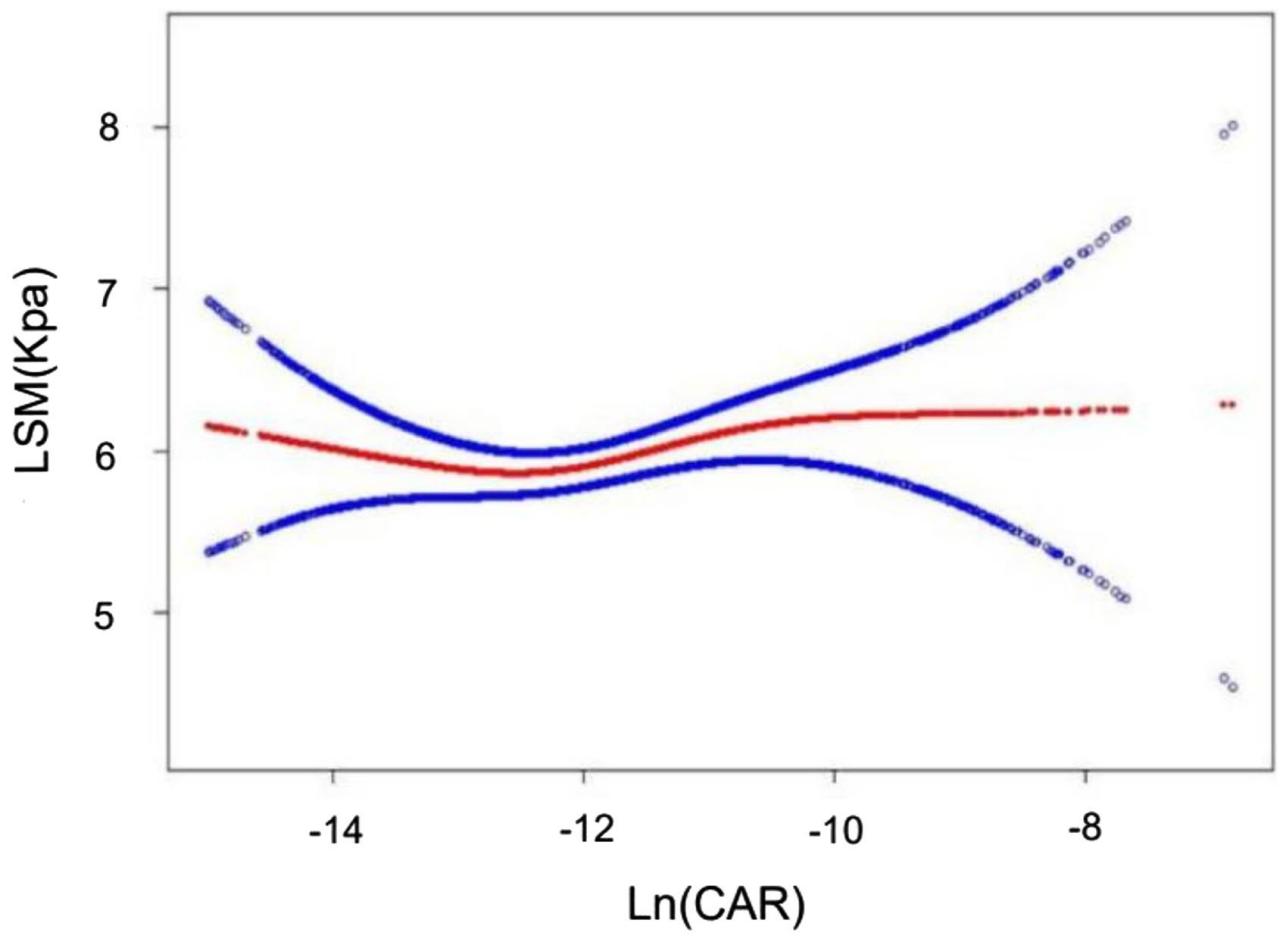
Nonlinear associations between Ln(CAR) and LSM. The solid red line represents the smooth curve fit between variables. The blue bands represent the 95% confidence interval from the fit.

Multiple regression revealed a weak relationship between Ln(CAR) and LSM in both the unadjusted and partially adjusted models. However, this association did not remain robust with all covariate adjustments (Table 6). Smooth curve fitting depicted the nonlinear relationship (Figure 4).

**Table 6:** Association between Ln(CAR) and LSM.

Ln(CAR)	Model 1 β (95% CI) *p* value	Model 2 β (95% CI) *p* value	Model 3 β (95% CI) *p* value
LSM(kPa)			
Continuous	0.602 (0.520, 0.684) <0.001	0.619 (0.581, 0.656) <0.001	0.032 (−0.084, 0.148) 0.589
Categories			
Tertile 1	Reference	Reference	Reference
Tertile 2	0.345 (0.101, 0.590) 0.006	0.272 (0.025, 0.520) 0.031	−0.315 (−0.606, −0.023) 0.034
Tertile 3	1.781 (1.534, 2.028) <0.001	1.798 (1.545, 2.050) <0.001	0.155 (−0.179, 0.489) 0.363
*p* for trend	<0.001	<0.001	0.282

Model 1: no covariates were adjusted. Model 2: Age, gender, and race were adjusted. Model 3: Age, gender, race, educational level, BMI, income-to-poverty ratio, moderate activities, smoking status, DM, hypertension, history of CVD, ALP, ALT, AST, total calcium, TC, TG, LDL-C, HDL-C, UC, and serum phosphorus were adjusted. NAFLD, nonalcoholic fatty liver disease; CAP, controlled attenuation parameter; LSM, liver stiffness measure; CAR, high-sensitivity C-reactive protein to albumin ratio.

## 4 Discussion

In this cross-sectional study including 7,404 participants, Ln(CAR) levels correlated with liver steatosis but not with fibrosis in NAFLD patients. Moreover, a nonlinear relationship exhibiting an inverted U-shaped curve was identified between Ln(CAR) and CAP, with a notable inflection point at (−9.594). The results suggested that Ln(CAR) functions as an independent risk factor for hepatic steatosis, independent of conventional risk factors when its value is less than −9.594. In addition, analysis of subgroups and interaction tests demonstrated a stronger significant relationship between Ln(CAR) and CAP in participants with a BMI ≥ 25.

To the best of the authors’ knowledge, no studies have previously been published on this topic. In the liver field, much of the focus of CAR in past research has been on predicting the prognosis or survival of patients with liver cancer or liver transplantation (13, 41–43). As reported by Haruki et al. (44), sustained elevation of the CAR is an important index for predicting adverse long-term outcomes among hepatocellular carcinoma patients after hepatic resection. Similarly, a study conducted in Japan involving 522 patients with unresectable hepatocellular carcinoma receiving lenvatinib treatment demonstrated a significant prognostic disparity between patients with low and high CARs. Compared with patients with lower CAR levels, patients with higher levels had markedly shorter median progression-free survival, implying that the CAR was a strong predictor of therapeutic outcomes in this cohort (45). In addition, a study from Germany revealed that the preoperative CAR was another reliable tool for predicting the perioperative risks of morbidity and mortality among patients receiving deceased-donor liver transplants (46).

Evidence from various studies underscored the significant role of inflammation in the pathophysiological progression of NAFLD. For example, lipotoxicity is considered a crucial pathological mechanism regarding the onset of NAFLD, which involves the activation of liver inflammatory mediators. To be specific, lipotoxicity triggers two classic molecular drivers of inflammation, the transcription factor nuclear factor-κB (NF-κB) and the NOD-like receptor pyrin domain containing 3 (NLRP3) inflammasome, and subsequently, both of which lead to the enhancing expression of pro-inflammatory cytokines like tumor necrosis factor-*α* (TNF-α), interleukin (IL)-6, and Il-1β (47). In addition to that, activated Kupffer cells in the liver secrete pro-inflammatory cytokines, which significantly contribute to the hepatic inflammatory process (48–51). In this context, Monocyte chemoattractant protein-1 (MCP-1) facilitates the migration of monocytes and various immune cells into the liver, exacerbating the inflammatory response and prompting hepatic stellate cell (HSC) activation (48, 49). HSCs further recruit inflammatory load, promote the secretion of inflammatory cytokines, and transdifferentiate into myofibroblasts, thus also acting as a pivotal contributor to liver fibrosis (48, 49, 52, 53). What is worse, in response to hepatocyte injury, recruited monocyte-derived macrophages can contribute to a self-perpetuating cycle of liver tissue damage in NASH-associated inflammation (54). In addition, liver sinusoidal endothelial cells (LSECs) accelerate the advancement of NASH through the secretion of inflammatory and fibrosis-inducing agents (55). Cappel et al. (56) demonstrated that inhibiting CAC flux by deleting pyruvate carboxylase, an anaplerotic node activated by acetyl-CoA, causes the induction of liver oxidative stress and inflammation. Our findings align with previous studies, highlighting a close correlation between inflammation and liver steatosis. Notably, hepatic steatosis results from a complex interplay among inflammation, nutritional status, and metabolic adaptation. Studies (57) have shown that inflammation plays a dual role in the development and progression of nonalcoholic fatty liver disease (NAFLD). Moderate inflammation promotes hepatic lipid accumulation, whereas excessive inflammation accelerates fat breakdown and impairs lipid retention. Additionally, evidence (58) suggests that albumin, a key regulator of fatty acid metabolism, facilitates lipid synthesis under moderate inflammatory conditions but becomes less effective under severe inflammation. Furthermore, sustained high-grade inflammation may drive metabolic adaptations, resulting in enhanced fat mobilization to meet the energy demands of immune responses and basal metabolism at the expense of lipid storage (59). These factors may underlie an inverted U-shaped nonlinear relationship between Ln(CAR) and CAP, rather than a simple linear association.

However, there is insufficient evidence to indicate an association with hepatic fibrosis. In accordance with a large multicenter cohort study in Italy and Finland, the data showed that 33% of patients with significant fibrosis, diagnosed through biopsy specimens at a single time point, lacked histological signs of nonalcoholic steatohepatitis (NASH) (60). Another analysis suggested that, while a significant proportion of NAFLD individuals exhibited simple steatosis, a subgroup of these individuals advanced to NASH, with only 20% developing cirrhosis (61). On top of that, the interaction between BMI and Ln(CAR) in this study suggested that Ln(CAR) may have a more significant effect on CAP by enhancing inflammatory response in individuals with higher BMI. Recent findings in the literature demonstrated that the systemic immune inflammation index (SII) and systemic inflammation response index (SIRI), as cost-effective and accessible markers of systemic inflammation, exhibited a positive correlation with obesity in American adults (62). Furthermore, another study from America supported the conclusion that inflammatory response is triggered early in adipose tissue expansion and during chronic obesity, leading to a permanent shift in the immune system toward a pro-inflammatory phenotype (63). Not only that, but evidence from a large prospective study revealed that lifestyle-induced weight loss substantially improves liver histology in NASH-confirmed patients via liver biopsy (64). Prior studies have identified that BMI can influence the activity of inflammatory markers. However, our study further revealed its moderating effect on the relationship between Ln(CAR) and CAP, underscoring the need for stratified analysis in assessing metabolic risk factors in populations with varying body compositions. In addition to BMI, other demographic variables such as age, gender, race, and additional laboratory tests and lifestyle variables may also exert influence on the association between Ln(CAR) and CAP and LSM. Moreover, the nonsignificant association between Ln(CAR) and LSM in the fully adjusted model might be attributed to the interaction of multiple factors.

There are two strong points of our study including a substantial sample and the appropriate application of the NHANES database. We gathered data on all patients from March 2017 to February 2020, covering the pre-pandemic period. Moreover, it is well established that the NHANES offers a significant advantage due to its utilization of a substantial, nationally representative sample. The extensive data available from NHANES, encompassing not only comprehensive demographic, socioeconomic, physical measurements, and lifestyle factors but also thorough laboratory testing and examination information, presents us with a valuable opportunity to control for various risk factors for CAR. Nevertheless, the shortcomings of the current study need to be mentioned. First, this analysis was cross-sectional, and therefore we cannot explicitly confirm causation. Second, despite including all reasonable covariates in the analysis to account for potential confounders, there remains the possibility of unmeasured covariates that could not be entirely ruled out. Third, transient elastography, rather than liver biopsy, was employed to evaluate the stage of hepatic steatosis and fibrosis. After all, transient elastography cannot be completely equivalent to liver biopsy. Finally, our study primarily focused on adult participants, with a smaller representation of a younger age group.

## 5 Conclusion

In conclusion, we demonstrate an inverted U-shaped relationship between Ln(CAR) and CAP risk within the U.S. demographic. Our results suggest that CAR may serve as a valuable diagnostic tool for NAFLD. Further prospective research is necessary to validate this conclusion.

## Data Availability

The datasets presented in this study can be found in online repositories. The names of the repository/repositories and accession number(s) can be found at: www.cdc.gov/nchs/nhanes/.

## Glossary

CRP: C-reactive protein
hs-CRP: High-sensitivity C-reactive protein
CAR: The ratio of high-sensitivity C-reactive protein to albumin
NCHS: National Center for Health Statistics
NHANES: National Health and Nutrition Examination Survey
NAFLD: Nonalcoholic fatty liver disease
NASH: Nonalcoholic steatohepatitis
VCTE: Vibration-controlled transient elastography
CAP: Controlled attenuation parameter
LSM: Liver stiffness measurement
ROC: Receiver operating characteristic
AUC: Area under the curve
SBP: Systolic blood pressure
DBP: Diastolic blood pressure
HBP: High blood pressure
COVID-19: Coronavirus disease-2019
BMI: Body mass index
LDL-C: Low-density lipoprotein cholesterol
HDL-C: High-density lipoprotein cholesterol
ALT: Alanine transaminase
ALP: Alkaline phosphatase
AST: Aspartate aminotransferase
TG: Triglyceride
TC: Total cholesterol
UC: Uric acid
CVD: Cardiovascular disease
DM: Diabetes mellitus
SII: Systemic immune inflammation index
SIRI: Systemic inflammation response index
