# Supplementary material for: An inverted U-shaped association between high-sensitivity C-reactive protein and the albumin ratio and hepatic steatosis and liver fibrosis: a population-based study
Source: Front Nutr. 2025 Apr 15;12:1534200. doi: 10.3389/fnut.2025.1534200 (PMC12037389; doi:10.3389/fnut.2025.1534200)
Supplement: Supplementary file 1 [file Table_1.docx]

**Supplemental** **Table1.** The best threshold, sensitivities, specificities, and area under the curve of Inflammatory markers for screening NAFLD in the general population

|  | **AUC** | **95% CI** | **Best threshold** | **Specificity** | **Sensitivity** |
| --- | --- | --- | --- | --- | --- |
| \| CAR \| 0.6895 \| 0.6841-0.6949 \| 0.00001 \| 0.6092 \| 0.7276 \| \| --- \| --- \| --- \| --- \| --- \| --- \| \| SII \| 0.5459 \| 0.5400-0.5518 \| 423.0588 \| 0.5066 \| 0.5660 \| | | | | | |

AUC: area under the curve; CAR: the high-sensitivity C-reactive protein to albumin ratio; SII: systemic immune-inflammation index.
